# Glutamine: a new strategy for targeted metabolic therapy in the tumor microenvironment

**DOI:** 10.1038/s41420-025-02767-4

**Published:** 2025-10-13

**Authors:** Haixu Lv, Xiao Han, Yuanshuang Yang, Jianfeng Shen, Yixuan Yin, Yu Liu

**Affiliations:** 1 Department of Surgery, Heilongjiang Provincial Hospital, Harbin, 150081 China; 2 Department of Pathology, School of Basic Medical Sciences, Harbin Medical University, Harbin, 150081 China; 3 Department of Magnetic resonance, Heilongjiang Provincial Hospital, Harbin, 150081 China

**Keywords:** Cancer metabolism, Cancer microenvironment

## Abstract

In the tumor microenvironment, glutamine has a profound impact not only on the growth, metabolism, metastasis, and invasion of tumor cells but also on the survival and function of immune cells and the behavior of nonimmune cells. Given the limited amount of glutamine in the tumor microenvironment, there is a competitive relationship between tumor and nontumor cells. Owing to the metabolic reprogramming of tumor cells, many nutrients, including glutamine, are necessary for tumor cells to maintain their rapid growth and high metabolic demand. Therefore, tumor cells are in a superior position to compete for glutamine. These findings provide solid theoretical support for targeting glutamine metabolism for anticancer therapy. This review summarizes the importance and necessity of glutamine for tumor cells and nontumor cells in the tumor microenvironment. According to the mechanism of action of glutamine in tumor cells and the regulatory mechanism of related signaling pathways, the currently developed anticancer drugs that target glutamine metabolism are categorized on a scientific basis, and the importance of basic medicine applied in clinical medicine is emphasized. This review not only provides anticancer information for clinicians but also brings hope to cancer patients.

## Facts

- Tumor cells have an absolute advantage in the competition for glutamine with non-tumor cells.
- Glutamine plays an important role in the growth and metabolism of tumor cells and the activation and polarization of non-tumor cells.
- Targeted therapy of glutamine metabolism is of great significance in the future clinical treatment of cancer.

## Introduction

Cancer is a complex ecosystem that includes both tumor cells and many nontumor cells and their interactions. Cancer metabolism likewise involves tumor cell metabolism and noncancer cell metabolism [1]. Cancer metabolism originally stems from aerobic glycolysis, also known as the Warburg effect, in cancer cells. Research has expanded cancer metabolism to also cover topics such as glucose, amino acid synthesis, fatty acids, and nucleotides [2]. During the process of cancer metabolism, cancer cells reprogram their metabolic pathways to maintain rapid proliferation and high energy requirements to ensure adequate nutrition and high metabolic demand, among which amino acid metabolism plays an important role in cancer metabolism [3]. Glutamine is an emerging star of amino acid metabolism and has attracted much attention. In recent years, numerous studies have shown that the effects of glutamine on the occurrence and development of tumor cells and the functions of nontumor cells should not be underestimated, thus providing theoretical support for the development of cancer therapies that target glutamine metabolism.

This review focuses on tumor cells and nontumor cells and the differences in glutamine metabolism between them. In this study, the competition between tumor cells and nontumor cells for glutamine in the tumor microenvironment (TME) is thoroughly analyzed, and the importance of glutamine for these two types of cells is explained in detail. In addition, this review provides an overview of glutamine and its molecular mechanisms of action in the various components of the TME, as well as a summary review of clinical research on glutamine metabolism as a therapeutic target. The aim of this review is to provide the latest information on cancer treatment and facilitate the development of new treatment strategies.

## Glutamine—an essential member of cancer metabolism

Bradley I. Reinfeld et al. used PET tracers to examine the uptake of glucose and glutamine by specific cell subsets in the TME and found that cancer cells take up more glutamine than glucose [4]. Glutamine has become an indispensable and important component of tumor cell growth and metabolism, providing fuel for a variety of metabolic pathways by participating in energy formation, redox homeostasis, macromolecular synthesis, and signal transduction in tumor cells [5]. A metabolomics analysis of pancreatic ductal adenocarcinoma and thyroid cancer revealed that glutamine consumption was extremely high in tumor tissues compared with nontumor tissues [6]. The serum glutamine concentration in colorectal cancer (CRC) patients is significantly lower than that in healthy people, which may also be due to the high uptake of glutamine by cancer cells to meet their high metabolic demand [7]. In addition, glutaminase and glutamine transporters are effective antitumor targets in triple-negative breast cancer because of their high uptake and dependence on glutamine [8]. An increasing number of studies have confirmed that the proliferation and metabolism of various cancer cells, such as non-small cell lung cancer, breast cancer and glioma cells, are highly dependent on glutamine [8, 9]. It can be concluded that cancer cells have a high affinity for glutamine. Specifically, studies on glutamine starvation in CRC cells revealed that, with prolonged glutamine starvation, the growth of CRC cells is reduced by approximately 40–50% compared with that of CRC cells with sufficient glutamine [10]. Glutamine, a metabolic component of essential amino acids, plays a key role in CRC growth, further highlighting the importance and necessity of glutamine in tumor cell metabolism.

Moreover, glutamine plays a pivotal role in the metabolism of nontumor cells. P. Newsholme et al. reported that glutamine is essential for the normal function of immune cells such as lymphocytes, macrophages, and neutrophils [11]. Glutamine may be involved in immune processes such as proliferation, antigen presentation, cytokine production and phagocytosis [11]. Fibroblasts (CAFs) constitute the largest group of nonimmune cells in the TME, and there is increasing evidence that CAFs can regulate glutamine levels in the TME and thus generate energy to promote the tricarboxylic acid cycle (TCA) cycle and supply energy for cancer cell growth [12]. Thus, glutamine plays an important role in the process of metabolism in tumor cells.

In the context of limited glutamine, various cell components in the TME compete for glutamine, and tumor cells can increase their uptake of glutamine due to their unique metabolic mode, thus providing certain competitive advantages. Therefore, the role of glutamine in the TME must be considered.

## The role of glutamine in the TME

The TME is a complex and dynamic cellular environment composed of tumor cells; immune cells such as macrophages and lymphocytes; nonimmune cells such as CAFs and tumor endothelial cells; and the extracellular matrix [13]. Glutamine is involved in a variety of metabolic processes in the TME; it provides necessary help for the growth, metabolism, and biosynthesis of tumor cells, immune cells, and nonimmune cells in the TME and also assists with the regulation and maintenance of the TME.

### Effect of glutamine on tumor cells

**Fig. 1:**
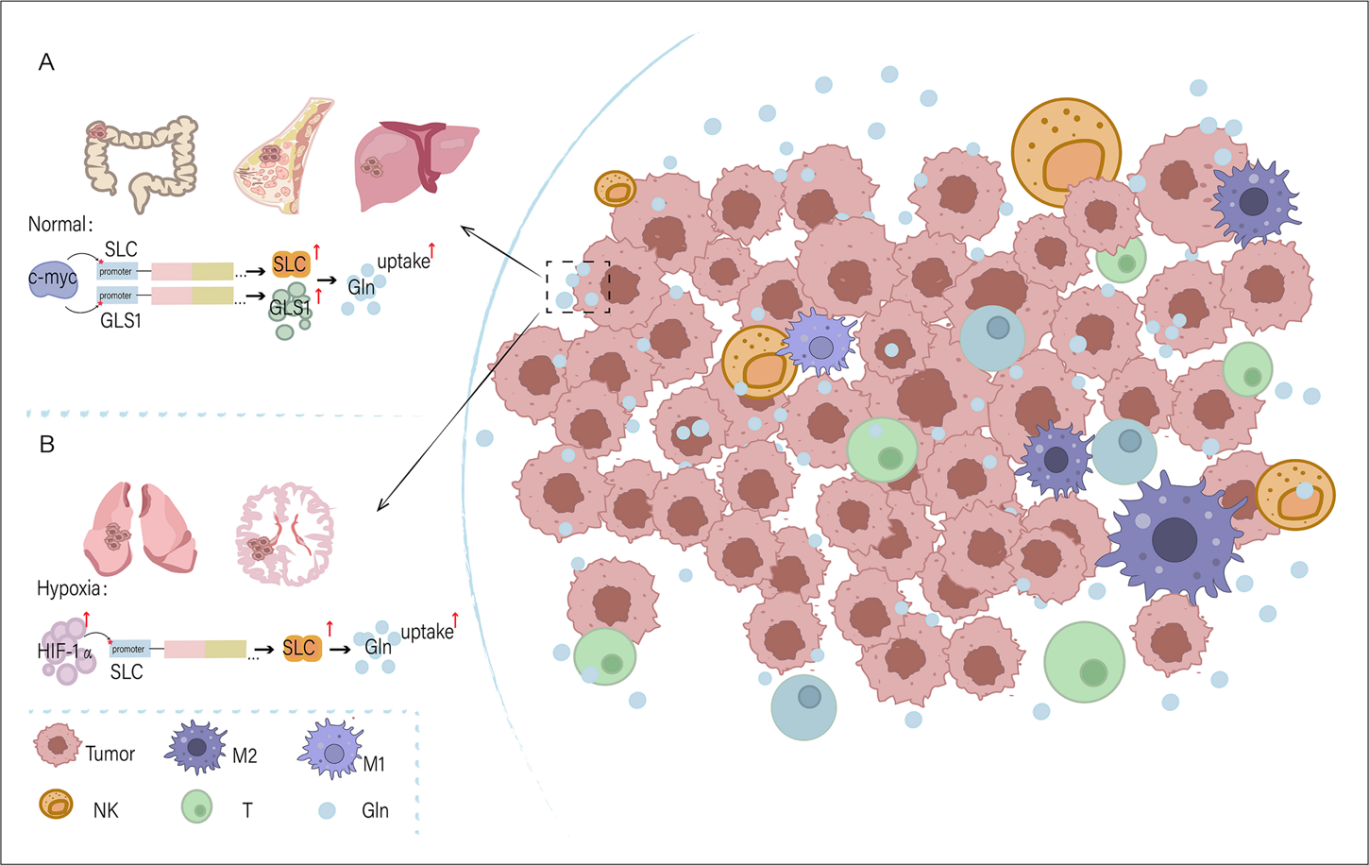
Glutamine addiction of tumor cells. In the tumor microenvironment, tumor cells are in a favorable position to compete with non-tumor cells for glutamine. **A** Glutamine is normally taken up by tumor cells. **B** Glutamine uptake by tumor cells under hypoxia.

**Fig. 2:**
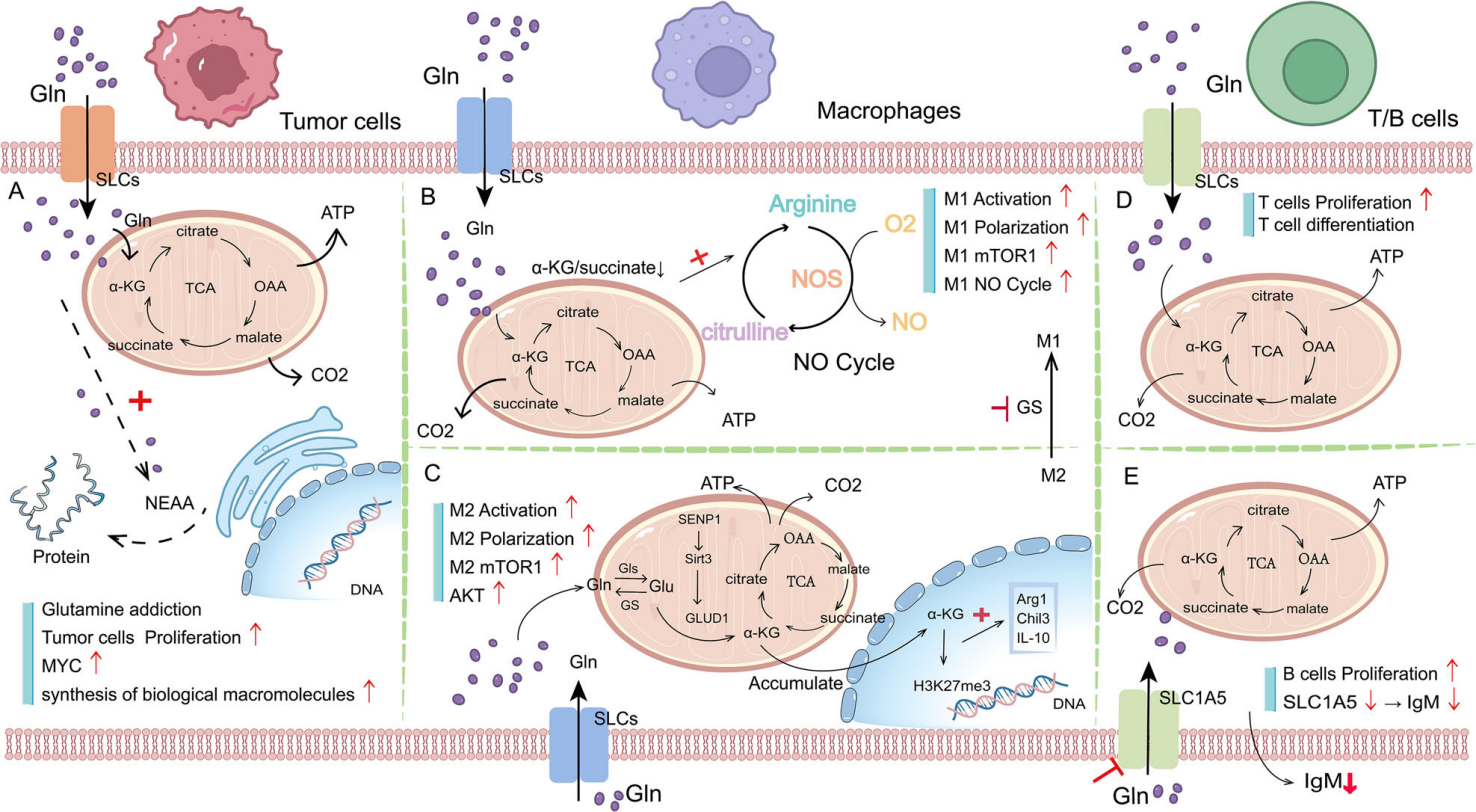
Effects of glutamine on cell growth and metabolism in tumor microenvironment. **A** Glutamine addiction, glutamine catabolism, and its influence on protein synthesis in tumor cells. **B**, **C** Catabolism of glutamine in macrophages and its effect on polarization of M1/M2. **D**, **E** Catabolism and function of glutamine in T/B lymphocytes. (Gln: glutamine).

In the TME, cancer cells exhibit excessive dependence on glutamine for proliferation and metabolic activities due to their unique metabolic pattern, known as “glutamine addiction”[14] (Fig. 1). The main feature of “glutamine addiction” is the increased transport of glutamine across the plasma membrane [15]. This phenomenon is due to the upregulation of the expression of glutamine transporters or enzymes by oncogenes such as c-MYC, which accelerate the uptake and metabolism rate of glutamine and then drive the TCA and biosynthetic processes [3, 16] (Fig. 2A). In addition, “glutamine addiction” varies significantly across cancer types. In CRC [17], breast cancer [18] and liver cancer [19], c-MYC upregulates the glutamine transporter by binding to the promoter region of the transporter and Glutaminase-1 (GLS1) and induces Glutaminase (GLS) expression at the mRNA and protein levels. This helps cancer cells take up more glutamine and supports the metabolic demands of cancer cells, thereby promoting glutamine uptake and metabolism by tumor cells and supporting the rapid proliferation and growth of tumor cells (Fig. 1A). In contrast, non-small cell lung cancer (NSCLC), glioblastoma [20] and other cancers have relatively low levels of intracellular glutamine metabolism under normal conditions, but under hypoxic conditions, the expression level of HIF-1α is significantly increased. HIF-1α promotes glutamine uptake by increasing the expression of glutamine transporters, such as SLC1A5. In addition, HIF-1α can promote the decomposition and utilization of glutamine by upregulating the expression of glutamine metabolic enzymes [21], thus strengthening its own growth metabolism (Fig. 1B). The mechanisms of glutamine addiction in different types of cancer cells differ, and these differences are caused by the different metabolic pathways of different tumor cells and the influence of the tumor microenvironment.

After a large amount of glutamine enters tumor cells, it is catabolized into glutamate and ammonia by GLS, and glutamate is catabolized by glutamate dehydrogenase or transaminase to provide a carbon source for the tricarboxylic acid cycle. In addition, approximately half of the nonessential amino acids required for protein synthesis in tumor cells are derived from Gln [22, 23]. The importance of glutamine for tumor cells can be seen.

### Effects of glutamine on immune cells

Gln is also extremely important for nontumor cells. Tumor-associated macrophages (TAMs) are among the most abundant cells in the TME and are usually M2-like macrophages. M2 macrophages can promote the proliferation of tumor cells, tissue repair, and the immune escape of tumor cells. Glutamine is essential for the activation and polarization of M2 macrophages. The ratio of α-KG produced by glutamine metabolism to succinic acid affects the activation of M2 macrophages. An increase in the α-KG/succinic acid ratio can promote the activation of M2 macrophages [24]. Abhishek K. Jha et al. reported that the UDP-GlcNAc pathway plays a key role in the polarization process of M2 macrophages and that glutamine is the main source of nitrogen in UDP-GlcNAc [25]. Thus, glutamine affects the physiological and functional regulation of M2 macrophages. In addition, the activation of SENP1-Sirt3 signaling leads to the accumulation of glutaminolytic α-KG in M2 macrophages, which enhances M2 macrophage polarization through the demethylation of Jmjd3 at H3K27me3, thereby promoting tumor cell growth and survival in the TME (Fig. 2C) [24, 26]. Glutamine synthetase (GS) can maintain the phenotype of M2 macrophages, and the inhibition of GS skews M2-polarized macrophages toward the M1-like phenotype [27]. Thus, in the TME, antitumor effects can be enhanced, and adverse effects such as immunosuppression and tumor metastasis can be reduced. The proinflammatory phenotype of M1 macrophages and other immune functions are also inseparable from the role of glutamine. In M1 macrophages, a low ratio of α-KG as a result of glutamine producing succinate can enhance their proinflammatory phenotype, which is beneficial for inhibiting the growth of tumor cells and enhancing their own immune function [24, 28] (Fig. 2B).

In addition to TAMs, the proliferation and metabolic activities of T lymphocytes and B lymphocytes within the TME are also critically dependent on glutamine. Upon activation, T lymphocytes exhibit increased expression of SLC7A5, leading to increased glutamine uptake. The absorbed glutamine is subsequently catabolized into the TCA cycle, providing essential energy for cellular functions [29] (Fig. 2D). Erikka L. Carr et al. demonstrated in vitro that T cells are highly sensitive to glutamine levels and that reducing the glutamine concentration inhibits T cell proliferation [30]. Glutamine catabolism promotes the synthesis of glutathione, thus influencing T cell differentiation [31]. B lymphocytes that produce antibodies and cytokines also depend on glutamine catabolism. The inhibition of the glutamine transporter SLC1A5 can regulate the production of immunoglobulin by B cells and inhibit the production of IgM [23, 32] (Fig. 2E). Therefore, glutamine affects a series of behavioral activities, such as the proliferation, metabolism, and activation and polarization of immune cells.

### Effects of glutamine on non-immune cells

The activity of nonimmune CAFs in the TME is also closely related to Gln. In the TME, ammonia secreted by cancer cells stimulates CAFs and subsequently triggers autophagy-related signals in CAFs. Ammonia uptake by CAFs also reduces mitochondrial viability. These two processes cause CAFs to secrete more glutamine into the TME, which is taken up by cancer cells and enhances the metabolic capacity and mitochondrial viability of those tumor cells [12]. Studies have demonstrated that ovarian cancer cells and lung adenocarcinoma cells promote self-proliferation by absorbing the Gln metabolized by CAFs [33, 34].

These findings highlight the importance and necessity of glutamine for the TME. In the TME, glutamine is involved in the proliferation and metabolism of tumor cells, immune cells and nonimmune cells; it also affects their immune function and signal transduction process and is closely related to their behavioral activities.

## The regulatory mechanism of glutamine in the TME

### Glutamine relies on transporters to enter tumor cells

**Fig. 3:**
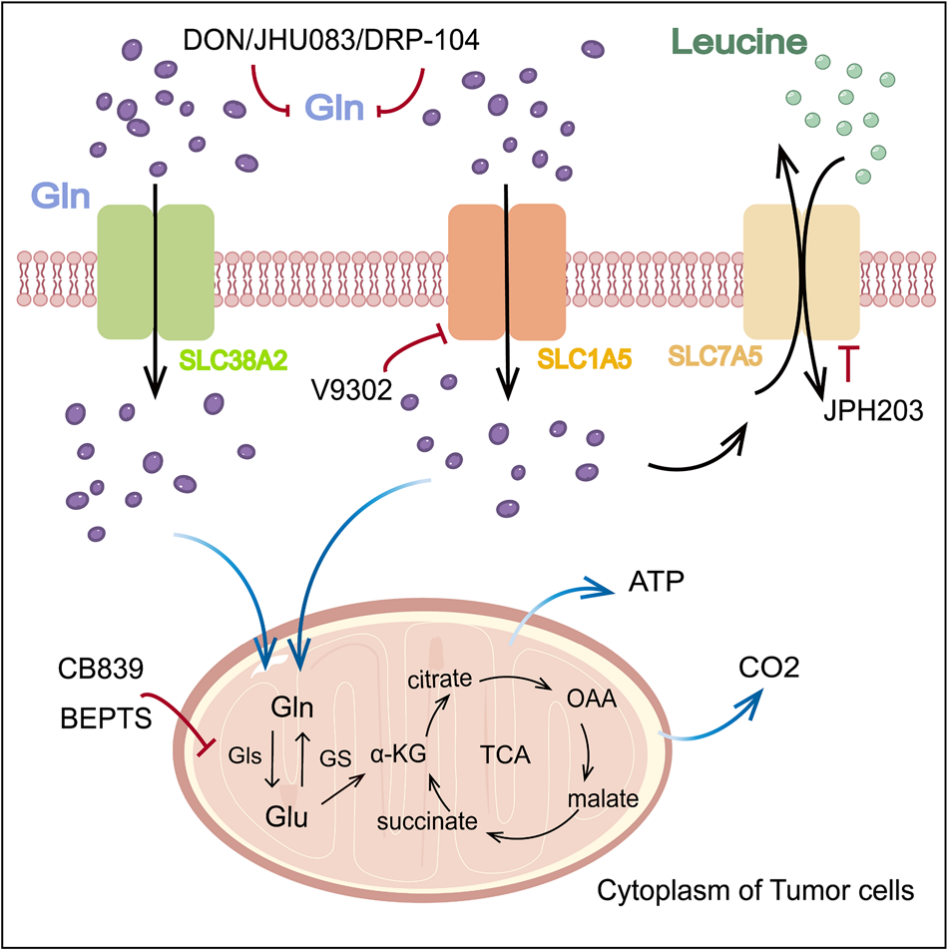
Targets and drugs for inhibiting glutamine metabolism. Glutamine enters tumor cells through transporters for catabolism, which is treated by two ways of targeting glutamine metabolism in cancer treatment, namely, targeting glutamine transporters and glutamine catabolism.

Glutamine is hydrophilic and must pass through certain selective transporters to enter the interior of tumor cells [35]. SLC proteins are a group of membrane cell and organelle transporters that control the uptake and efflux of various molecules and compounds [35]. SLC1A5, SLC7A5, and SLC38A2, as glutamine transporters (Fig. 3), can transport glutamine into tumor cells to drive metabolic processes such as the TCA cycle and REDOX balance, and the upregulated SLCs in cancer cells also increase the glutamine supply to tumor cells [36].

SLC1A5 has a strong affinity for glutamine. This affinity is determined by the structural characteristics and substrate specificity of the SLC1A5 gene, particularly in acidic environments, which ensures the growth and metabolism of cancer cells more effectively [12]. SLC1A5 is highly expressed in a variety of solid tumors, including TNBC, lung cancer, and ovarian cancer [37, 38]. Using in vitro and in vivo experiments combined with a clinical gene expression analysis of TN breast cancer patient samples, M. van Geldermalsen et al. found that highly expressed ASCT2 mediates glutamine uptake by basal-like TN breast cancer cells to maintain mTORC1 signaling, cell growth, and cell cycle progression [37]. In NK cells, the upregulation of SLC1A5 expression drives the production of IFN-γ factors, thereby enhancing antitumor and immune surveillance effects [39]. During the activation of naive T cells to mature T cells, SLC1A5 promotes the rapid uptake of glutamine by T cells to provide energy and biosynthetic demand for T cells [40].

SLC7A5 acts as a mandatory exchanger to mediate the efflux of leucine and glutamine from the cell through mutual coupling with SLC1A5 [41]. Experiments with colorectal tumors in mice revealed that SLC7A5 deletion significantly reduced the number of tumors in the small intestine and colon, suggesting that SLC7A5 promotes tumor occurrence and development [42]. The promoter of SLC7A5 can bind to the transcription factor HIF-2α to increase the activity of mTOR1 to support the proliferation of tumor cells when the TME is hypoxic and nutrient deficient [41, 43]. In addition, SLC7A5-mediated amino acid influx is essential for the production of the inflammatory cytokine IL-1β by monocytes and macrophages [44]. Therefore, SLC7A5 may be a potential therapeutic target for a variety of inflammatory diseases.

SLC38A2 is a Na+ ion-dependent neutral amino acid transporter belonging to the SLC38 family of transporters that drives glutamine flow into cells [45]. In TNBC, SLC38A2 expression is upregulated to promote glutamine dependence and confers oxidative stress resistance [46]; thus, SLC38A2 transporters can be potential targets in TNBC therapy. Furthermore, genetically engineered mouse models revealed that tumor cells and cDC1s compete for glutamine uptake through SLC38A2 to regulate antitumor immunity [47]. Therefore, inhibiting the SLC38A2 transporter is a possibility for cancer treatment. DC cells can effectively absorb and present tumor antigens by expressing SLC38A2, which is essential for maintaining the immune response of antigen-specific CD8 + T cells [47].

The functional difference in SLC protein expression between tumor cells and immune cells is particularly obvious. Tumor cells use proteins such as SLC1A5 to transport various amino acids and other nutrients and increase the uptake and predation of nutrients by highly expressing SLC transporters to strengthen their own metabolic reprogramming. In addition to using SLC proteins to take up nutrients, immune cells participate in the regulation of immune cell differentiation and polarization and affect the activity and function of immune cells. The difference in the effects of SLC protein expression on tumor cells and immune cells suggests that targeting the metabolism of tumor cells, inhibiting glutamine uptake by tumor cells, reversing the dominant position of glutamine competition in tumor cells in the TME, and activating immune function to kill tumor cells may be new strategies to explore.

### Regulation of glutamine-related metabolic genes and signaling pathways

In the TME, the entry of glutamine into tumor cells depends on transporters; moreover, it is regulated by carcinogenic or tumor suppressor genes and related pathways.

c-MYC is a common human oncogene and is actively involved in glutamine metabolism in cancer cells. MYC can bind to the promoter elements of ASCT2 and SN2 transporters, promote the synthesis of glutamine transporter mRNAs, upregulate the expression of glutamine transporters in cancer cells, and thus promote the growth and metabolism of tumor cells [16]. Similarly, MYC binds directly to the promoter elements of glutamine transporters such as ASCT2 and SN2 in T cells to promote their mRNA synthesis, thereby increasing glutamine uptake and metabolism and supporting the activation and functional maintenance of immune cells [17]. Although the mechanisms by which MYC regulates glutamine metabolism are similar in tumor cells and immune cells, there are significant differences in the metabolic pathways of MYC between these cell types. In tumor cells, MYC-driven glutamine metabolism promotes tumor growth, drug resistance and immune escape [48], whereas MYC-regulated glutamine metabolism in immune cells supports the activation, proliferation and cytokine secretion of effector T cells and NK cells and enhances the immune response and other immune functions [49]. This difference reflects the different adaptive needs of glutamine metabolism between tumors and the immune system and provides a strategic basis for cancer therapies that target metabolism.

Mutant KRAS alters the basal metabolism of cancer cells and supports cancer cell proliferation by increasing glutamine utilization [42]. Mutations in KRAS lead to the upregulation of the expression of the SLC7A5, SLC38A2, and SLC25A22 transporters in cancer cells, thereby promoting glutamine uptake [50]. After the conversion of ingested glutamine to glutamate, it actively participates in the TCA cycle. Other participants in the TCA cycle are also more active in KRAS-mutant tumors, particularly in pancreatic ductal adenocarcinoma (PDAC), where the increased expression of GOT1/2 supports glutamine metabolism while producing NADPH to maintain REDOX balance [50]. Palanivel Kandasamy and others introduced KRAS mutations into CRC cells in culture medium containing different concentrations of glutamine and reported that compared with wt KRAS CRC cells, mutant KRAS CRC cells had a significantly lower proliferation rate and a lower glutamine concentration; that is, mutant KRAS CRC cells have a greater requirement for glutamine [51]. Moreover, mutant KRAS CRC cells were more dependent on the glutaminolysis pathway for energy supply than were wt KRAS CRC cells [52]. Therefore, drugs targeting this metabolic pathway may be more effective in KRAS-mutant CRC.

p53 is a tumor suppressor gene that prevents carcinogenesis by recognizing cell damage and inducing apoptosis. In recent years, the p53-inducible gene GLS2 has been found to regulate glutamine metabolism and ROS levels in cancer cells and promote antioxidant defense by controlling the GSH/GSSG ratio [53]. Therefore, p53-mediated GLS2 could be a potential target for tumor therapy.

**Fig. 4:**
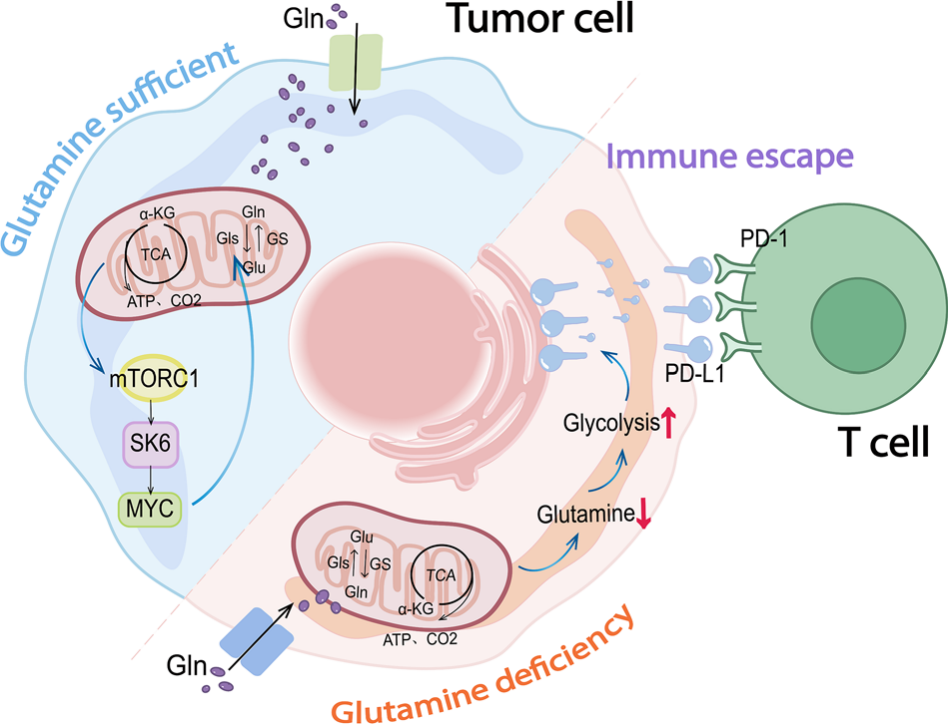
Effects of glutamine on signaling pathways and genes associated with tumor cells. Glutamine sufficient: glutamine metabolism in tumor cells activates the mTORC1 pathway and promotes the expression of GLS, thereby promoting glutamine catabolism. Glutamine deficient: tumor cells maintain their activities through metabolic compensation-mediated enhancement of glycolysis and immune escape induced by PD-L1 up-regulation.

In addition to gene regulation, the mTOR pathway is closely related to glutamine metabolism. P. Nicklin et al. demonstrated that glutamine can directly act on the MTON-S6K1-S6 pathway, and their results revealed that tumor cells take glutamine into the cell through SLC1A5, glutamine supplies energy to the cell and then effluxes through SLC7A5A/SLC38A2. The efflux process promotes the entry of leucine and arginine, thereby activating the mTORC1 signaling pathway [54]. Guokai Yan et al. verified that activated mTORC1 phosphorylates S6K1 at Thr389, causing it to undergo a conformational change and become activated. S6K1 subsequently phosphorylates Ser235, Ser236, Ser240, and Ser244 of the S6 protein, promoting the activation of S6 and ultimately enhancing ribosome [55, 56]. In addition, when mTORC1-S6K1 is activated, S6K1 can phosphorylate eIF4B at Ser422 and promote the formation of the mRNA translation initiation complex. Phosphorylated eIF4B further inhibits miR-23a/b expression by promoting the translation of myc mRNA and increasing the protein level of myc, which acts as a transcription factor. Thereby reducing the inhibition of GLS and promoting glutamine hydrolysis (Fig. 4). This provides a continuous supply of raw TCA materials for tumor cell growth and metabolism [16, 57]. In contrast, due to the competition of tumor cells for glutamine in the TME, glutamine deficiency in immune cells leads to the inhibition of mTOR activation in immune cells [23], which then weakens TCR signaling in T cells, reduces Th1/Th17 differentiation. B-cell proliferation and antibody production are thus affected [58]. Tumor cells occupy a dominant position in the competition for glutamine with immune cells. While enhancing their own metabolic reprogramming, tumor cells weaken the function of immune cells and induce the immune escape of tumor cells. These findings suggest that targeted regimens from multiple dimensions, such as metabolic intervention, immune microenvironment remodeling, combined treatment strategies, and collaboration with other therapies, must be designed to achieve precise cancer treatment.

The activation of programmed death-1 (PD-1)/its ligand programmed death ligand 1 (PD-L1) is one of the main mechanisms of tumor cell immune escape. Their interaction can inhibit the proliferation of immune T cells, leading to tumor cell immune escape [59]. PD-1/PD-L1 expression can be regulated by multiple mechanisms in the TME, including glutamine metabolism [60]. The inhibition of glutamine metabolism in tumor cells leads to the metabolic compensation-mediated enhancement of glycolysis and immune evasion induced by PD-L1 upregulation (Fig. 4), significantly limiting the therapeutic effect of glutamine inhibitors. In bladder and kidney cancers, tumor cells can activate EGFR signaling when glutamine-deficient tumor cells fail to proliferate normally. After EGFR, Ras, Raf, and MEK1/2 are activated through a series of cascade reactions, ERK1/2 is activated. The signal is subsequently transmitted to the nucleus by the c-Jun, ELK-1 and STAT3 transcription factors, and different transcription factors bind to different sites of the PD-L1 promoter to form multiprotein complexes. Subsequently synergizing with the transcription level of PD-L1 in tumor cells. PD-1 can upregulate the expression of PD-L1 in tumor cells and ultimately inhibit the proliferation of T cells and reduce immune function by binding to its ligand PD-L1, thereby facilitating tumor cell immune escape [61–64], this is the mechanism by which tumor cells evade immune cells with the help of PD-1 in the absence of glutamine. This mechanism provides a new way to intervene in the immune escape of tumor cells.

These findings clarify the competitive relationship between glutamine in tumor cells and nontumor cells, which may be a potential target for future clinical treatment; thus, glutamine plays a role in tumor immunotherapy that cannot be ignored.

## Glutamine metabolism inhibition-related targets and clinical research

### Glutamine transporter inhibitors

As the mechanism of glutamine metabolism in the TME has been continuously explored and revealed, the potential of its related targets in tumor treatment has also received clinical attention. The aforementioned glutamine transporters are promising drug targets that can inhibit the uptake of glutamine by tumor cells from the source, block the nutritional and energy sources of tumor cells, and thus weaken the advantage of tumor cells in glutamine competition (Fig. 3) and (Table 1).

**Table 1:** Relevant drugs targeting glutamine metabolism for cancer treatment (Query using ClinicalTrials. gov).

Target	Drug	Cancer type	NCT	Clinical Trial Phase	Objective response rate	Combination therapy
**SLC1A5**	V9302	HNSCC^69^	—	—	—	—
	V9302	Breast Cancer	—	—	—	Standard chemotherapeutic drugs^70^
	V9302	UVM	—	—	—	NPs: HDACi, MS-275^71^
	V9302	Pancreatic Cancer	—	—	—	NPs: BAY876^72^
**SLC7A5**	JPH203	TNBC	—	—	—	Anti-PD1 antibody^8^
**GLS**	CB-839	MDS	NCT03047993	Phase Ib/II Completed	70%	AZA, Azacitidine, Nivolumab, TAK228, Capecitabine, low dose dexamethasone pomalidomide^73–76^
		AML	NCT02071927	Phase I Completed	The results have not yet been published.	
		MM, ccRCC, NSCLC	NCT02771626	Phase I/II Completed	MM: The results have not yet been published, ccRCC: 50%, NSCLC: 19%	
		NSCLC	NCT04250545	Phase I Dose-Escalation Phase	68.2%	
		PIK3CA-mut CRC	NCT02861300	Phase I/II Completed	26%	
		CRC	NCT03875313	Phase I/II Terminated	The results have not yet been published.	
	IPN60090	OC NSCLC	NCT03894540	Phase I Terminated	The results have not yet been published.	Venetocla, CB-839, TAK228^77,78^
	Telaglenastat	KRAS-mutated NSCLC^54^	__	—	—	
**Glutaminase**	DRP-104	FLC	NCT06027086	Phase Ib/II Ongoing		Durvalumab
		NSCLC	NCT04471415	Phase I/II Ongoing		
	JHU-083	Lung Tumor^79^	—	—	—	—
**MYC**	MYCi975	HNSCC	—	—	—	CB839
	10074-G5	Prostate Cancer	—	—	—	DON

V9302, a competitive antagonist that targets transmembrane glutamine transport, blocks the glutamine transport process in tumor cells by efficiently and selectively binding to the glutamine transporter SLC1A5 [65]. In breast cancer, V9302 promotes autophagy by regulating the accumulation of ROS. In combination with standard chemotherapy drugs or anti-PD-1 immunotherapy, V9302 has better anticancer potential in inhibiting tumor growth, reversing tumor drug resistance, and enhancing the immune response [66, 67]. This synergistic effect provides new strategies and ideas for the treatment of breast cancer and other cancers. A recent study developed a ROS-responsive nanodelivery system that codelivers HDACi, MS-275, and V9302 to ensure nanoparticle accumulation at the tumor site of uveal melanoma. When accumulated nanoparticles detect high levels of ROS in the TME, the therapeutic drug is released, which is more precise and efficient [68].

JPH203 is an inhibitor of SLC7A5 and is a compound designed on the basis of the conformational relationship of the SLC7A5 ligand; it has an extremely high affinity for SLC7A5 and has not shown obvious toxicity in preclinical studies. JPH203 is used to treat TNBC by blocking SLC7A5, and the combination of JPH203 with an anti- PD-1 antibody can improve its therapeutic effect, which represents a new method for the treatment of TNBC [8].

### Glutamine catabolism inhibitors

In cancer treatment, in addition to the inhibition of glutamine transporters, increasing attention has been given to enzymes related to glutamine catabolism. Glutaminase can be targeted with drugs to inhibit the breakdown of glutamine in cancer cells to supply energy and hinder the biosynthesis process to achieve antitumor effects (Fig. 3) and (Table 1).

As a glutaminase inhibitor, CB-839 is highly sensitive to esophageal squamous cell carcinoma (ESCC), which is highly dependent on glutamine metabolism for energy, providing a new possibility for the treatment of ESCC [69]. Similarly, the treatment of lung cancer cells with CB-839, which reduces glutaminase activity in tumors, also results in signs of reduced tumor growth [70]. Zhiyan Li and others built new nanocomposites on the basis of PDT. The photosensitizers and CB-839, after self-assembly in the tumor cell membrane, were encapsulated in the same targeted therapy for stomach cancer, effectively enhancing the antitumor effect [71]. In addition, the glutaminase inhibitor IPN60090 can be combined with CB839 in the treatment of Myelodysplastic Syndromes (MDS) and Acute Myeloid Leukemia (AML), and with increasing doses of CB839 and IPN60090, the levels of intracellular NADPH and NADP+ are reduced, thereby inhibiting the proliferation of MDS and AML cells [72].

DON is a glutamine antagonist that extensively inhibits glutamine metabolism and inactivates a variety of glutamine-metabolizing enzymes by competing for covalent binding to the active site of glutamine [73]. In PDAC, DON and its prodrug DRP-104 were found to arrest tumor proliferation and reduce metastasis in various mouse models [6]. The combination of JHU-083, which is also a prodrug of DON, was found to significantly reduce tumor cell size without inducing significant toxicity when it was used in lung tumor models [74]. Treatment with JHU-083 for lung cancer can not only inhibit the growth of tumor cells but also prevents unnecessary suffering in patients.

In response to the oncogene c-Myc upregulating GLS1 expression to promote glutamine metabolism in tumor cells, the MYC inhibitor MYCi975 has been found to target the MYC protein to treat cancer. The use of MYCi975 in HNSCC was found to significantly inhibit the proliferation, colony formation, and glutamine consumption of tumor cells [75]. The combination of MYCi975 and CB-839 was more effective at reducing c-Myc levels and inhibiting tumor growth without significant toxic effects. The combined treatment of MYCi975 and CB-839 almost completely inhibited lymph node metastasis, and the combined treatment also more strongly inhibited tumor metastasis than either inhibitor alone [75]. Therefore, the combination of the MYC inhibitors MYCi975 and CB-839 is a better strategy for the treatment of HNSCC.

The upregulation of the expression of glutamine transporters and increased expression of GOT1/2 in the TCA cycle in KRAS-mutant tumors are promising targets for the treatment of such cancers. The inhibition of the SLC7A5, SLC38A2 and SLC25A22 transporters may inhibit the uptake of glutamine in KRAS-mutant tumor cells, thereby inhibiting the growth and metabolism of tumor cells and achieving therapeutic effects [50].

The constructed PD-L1-targeted metabolism and immunomodulator (PMIR) is used to regulate metabolism in the TME to improve immunotherapy. PMIR inhibits glutamine metabolism in tumor cells, thereby increasing glutamine levels within the TME and thereby improving the function of immune cells. Concomitant PD-L1 upregulation in tumor cells can also be blocked by PMIR. Together, these effects lead to the immunogenic cell death of tumor cells and alleviate PD-L1-mediated immune evasion, which further remodels the immunosuppressive TME and triggers a robust immune response, effectively inhibiting bilateral tumor progression and metastasis [76]. This treatment offers a reasonable strategy to overcome the barrier of glutamine inhibition to facilitate existing clinical treatments.

These glutamine inhibitors have shown promise and potential as antitumor agents by inhibiting glutamine transporters and glutaminase and glutamine metabolism and have shown exciting results in clinical trials. However, we must continue to explore and improve our understanding of the mechanism of glutamine inhibitors.

## Discussion

Glutamine is an indispensable energy source in cancer cell metabolism. Therefore, the related targets of glutamine metabolism have important application prospects when designing clinical drugs for cancer therapy. The key targets, such as GLS, glutamine transporters, and related metabolic pathways, are mentioned above. However, when glutamine metabolism is targeted with a single treatment, tumor cells will activate other signaling pathways or strengthen glutamine metabolism reprogramming to meet their energy demand, resulting in different degrees of drug resistance in tumor cells, thus restricting targeted glutamine metabolism therapy.

The mechanisms of tumor resistance to glutamine-targeted therapy involve multiple levels, including changes in the status of glutamine transporters and the effects of other metabolites in the TME on glutamine metabolism. The glutamine transporter SLC7A8 on the membrane of tumor cells plays an important role in the process of drug resistance. SLC7A8 can activate the mTORC1 signaling pathway, increase glutamine uptake and glutathione synthesis, and further aggravate the drug resistance of tumor cells [77]. Therefore, it is not enough to target one transporter alone in cancer treatment. The combination of the mTORC1 inhibitor Everolimus to block the mTORC1 signaling pathway and reduce the uptake of glutamine by tumor cells from the extracellular environment may be the key to solving this problem. In addition, changes in metabolites in the tumor microenvironment are important causes of drug resistance. Studies have shown that when the cystine content in the TME increases, the dependence of tumor cells on glutamine metabolism can be reduced through cystine/glutamate antiporters; that is, the entry of cystine may provide an alternative metabolic pathway to reduce the dependence of cells on glutamine. When the GLS inhibitor CB-839 was used, the sensitivity of cells to this inhibitor decreased, resulting in drug resistance [78]. Therefore, blocking cystine/glutamate transporters and synergistically inhibiting glutamine metabolism may be an effective strategy for reversing tumor drug resistance and improving therapeutic effects. The drug resistance mechanism of glutamine-targeted therapy is complex and involves metabolic adaptability, microenvironment regulation, and compensatory pathway activation. In view of these mechanisms, combination therapy strategies can overcome drug resistance through multitarget synergy, regulating the TME, overcoming redox homeostasis, improving drug efficacy, and effectively reversing drug resistance.

The combination of targeted glutamine metabolism and immune checkpoint blockade is also an important strategy to overcome tumor drug resistance. Targeted metabolism combined with immune checkpoint PD-1/PD-L1 inhibitors can block inhibitory signal transmission between tumor cells and T cells to relieve the functional inhibition imposed by tumors on T cells, restore the recognition and killing functions of T cells to tumor cells, and reactivate the immune response of T cells. The ability to increase the body’s immune surveillance and then achieved for the purpose of cancer treatment [79, 80].

From a clinical perspective, patients can be divided into different subgroups on the basis of differences in glutamine metabolism across different cancer types, which has important application prospects in cancer prevention, screening, and treatment. Different tumor cells have different degrees of “glutamine addiction”, and this difference can be used as an important basis for patient stratification. Metabolic imaging technology can identify patients with tumors with abnormal glutamine metabolism [16] to provide a basis for patients to target glutamine metabolism in cancer treatment. In addition, the expression levels of the glutamine transporters SLC1A5 and SLC7A8 can be used as biomarkers to predict the response of tumors to glutamine-targeted therapy [81]. By detecting the expression levels of these transporters, a patient group that is more sensitive to glutamine-targeted therapy can be identified. In addition, the degree of immune cell infiltration in the tumor microenvironment affects the efficacy of glutamine-targeted therapy. Therefore, in the process of clinical diagnosis and treatment, cancer cells can be targeted more precisely according to individual differences in glutamine metabolism in tumor cells. This precision medical strategy can not only improve the therapeutic effect and reduce the unnecessary use of drugs but also reduces the risk of side effects in patients and improves their quality of life.

With further research on the mechanism of glutamine metabolism and the discovery of more related biomarkers, a more complete patient stratification system and a more personalized plan for cancer treatment can be established. Moreover, such studies will further promote the research and development of drugs related to glutamine metabolism inhibition and thus provide more treatment options and hope for cancer patients.
